# Natural deep eutectic solvent-ultrasound assisted extraction: A green approach for ellagic acid extraction from *Geum japonicum*

**DOI:** 10.3389/fnut.2022.1079767

**Published:** 2023-01-09

**Authors:** Zhao Yang, Shi-Jun Yue, Huan Gao, Qiao Zhang, Ding-Qiao Xu, Jing Zhou, Jia-Jia Li, Yu-Ping Tang

**Affiliations:** Key Laboratory of Shaanxi Administration of Traditional Chinese Medicine for TCM Compatibility, State Key Laboratory of Research and Development of Characteristic Qin Medicine Resources (Cultivation), Shaanxi Key Laboratory of Chinese Medicine Fundamentals and New Drugs Research, Shaanxi Collaborative Innovation Center of Chinese Medicinal Resources Industrialization, Shaanxi University of Chinese Medicine, Xi’an, Shaanxi, China

**Keywords:** *Geum japonicum*, natural deep eutectic solvents, ellagic acid, response surface methodology, ultrasound assisted extraction

## Abstract

**Introduction:**

In China and other Asian nations, *Geum japonicum* (GJ) is used as functional vegetables or as a type of folk medicine. Ellagic acid (EA) is one of the main active ingredients in GJ and has been utilized in food, cosmetics, and medicinal goods worldwide. Natural deep eutectic solvents (NADESs) have gradually replaced organic solvents for efficient extraction of plant-derived active compounds due to its environmental protection, low toxicity, low solubility, reusability, etc.

**Methods:**

NADES with the highest EA yield was selected and the extraction conditions were optimized by response surface methodology (RSM), the antioxidant activity of NADES extract was determined, and finally Fourier transform infrared (FT-IR) and scanning electron microscopy (SEM) were used to explain the mechanism for the increase of EA yield in GJ.

**Results:**

In this work, several NADESs were tailored for the ultrasound assisted extraction (UAE) of EA from GJ, among which choline chloride-oxalic acid (ChCl:Oa) was the most effective. In optimal conditions, ChCl:Oa extract produced higher EA yields than common organic solvents including methanol, ethanol, and acetone. *In vitro* antioxidant experiments showed that ChCl:Oa extract had stronger DPPH radical scavenging ability than other solvent extracts. Mechanically, FT-IR results indicated that ChCl:Oa could form a hydrogen bonding with EA, which enhanced the stability of EA. Meanwhile, ChCl:Oa-UAE treatment could destroy the tissue structure of GJ, thereby improving EA yield.

**Discussion:**

In conclusion, these results imply that the ChCl:Oa-UAE method might be an environmentally friendly approach for extracting EA from GJ.

## 1. Introduction

Ellagic acid (EA), a natural polyphenol dilactone and a dimeric derivative of gallic acid (1), has been utilized in food, cosmetics, and medicinal goods all over the world (2). EA is frequently utilized as a natural antioxidant since recent pharmacological and clinical investigations have demonstrated its potent capacity to scavenge free radicals, decrease oxidative stress, and increase the activity of antioxidant enzymes (3). In China and other Asian nations, *Geum japonicum* (GJ, “Lanbuzheng” in Chinese) is employed as functional vegetables or as a type of folk medicine (4). Clinical evidence has shown that GJ has some beneficial effects that are anti-myocardial ischemia and anti-hypertensive (5, 6). GJ contains a variety of chemical components, including tannins (primarily EA), terpenes, phenylpropanoids, and others (7). As a result, using EA from GJ in medicine and healthcare products could provide an alternative source of EA and increase the availability of GJ.

Organic solvents such as methanol, ethanol, and acetone have traditionally been used to extract EA from GJ (8). They can cause significant environmental pollution due to their flammability, volatility, and toxicity. If EA extracted by organic solvents is used in cosmetics, food, or pharmaceuticals, it is likely to be harmful to humans. There has been a lot of interest in EA extraction using a green extraction solvent that is acceptable in the chemical, food, and pharmaceutical industries (9). Meanwhile, another crucial factor to consider is vastly increasing EA yield with the appropriate extraction process. Traditional extraction techniques, including heating agitation extraction, heating reflux extraction, and microwave extraction, are incapable of preventing the molecular properties’ alteration of EA and yield loss caused by thermal degradation, ionized hydrolysis, or oxidation during the extraction process (10). Therefore, it is vital to create an effective and green method for extracting EA from GJ.

The use of safer solvents and additives is one of the six principles of green extraction, and the choice of appropriate solvents in the extraction procedure is extremely crucial (11). Natural deep eutectic solvents (NADESs) are solvents composed of hydrogen bond acceptors (HBA) and hydrogen bond donors (HBD) in a specific molar ratio, which are typically derived from naturally occurring, comparatively cheap, and widely available components (e.g., choline chloride, sugars, amines, alcohols, carboxylic acids) (12–14). NADESs have gradually replaced organic solvents for the efficient extraction and separation of alkaloids, flavonoids, polysaccharides, and phenolic acids in medicinal plants due to their good physical and chemical properties such as environmental protection, ease of preparation, low toxicity, low volatility, low solubility, and good reusability (15–21). Therefore, the idea of using NADESs as extraction solvent to extract EA from GJ seems to be feasible.

Ultrasound assisted extraction (UAE) is a fantastic approach to speed up extraction, reduce extraction time, and increase the yield of target compound (22, 23). The cavitation phenomena and mixing effects that cause the exudation of cell contents, accelerate the mass transfer rate between the plant matrix and solvent, and increase extraction efficiency are the fundamental reasons of the improved heat and mass transfer supplied by ultrasound (24, 25). Thus, a green and effective extraction of EA from GJ may be achieved by combining NADES, a green solvent, with UAE.

Response surface methodology (RSM) is a statistical experimental method for modeling functions of continuous variables. Specifically, RSM evaluates the various factors and their interactions that influence the experimental process, represents the functional relationship between the various factors with graphs, and provides the optimal experimental conditions (26). RSM requires fewer experimental groups, saves material as well as human resources, and is increasingly used for the optimization of extraction process parameters (27). With regard to Box-Behnken design (BBD), it is one of the commonly used RSM for explaining experimental results as it requires fewer experiments than other methods when the experimental factors are three or more (28). Therefore, BBD was chosen to optimize the extraction process of EA from GJ in this study.

To the best of our knowledge, no earlier reports of the NADES-UAE approach for extracting EA from GJ exist. The purpose of this study was to examine the efficiency of EA extraction from GJ by customizing NADES as a green solvent and optimizing the procedure by investigating the impact of various parameters which include water content, extraction time, solid-liquid ratio, extraction temperature, and extraction power. Additionally, we examined the antioxidant ability of NADES extracts to scavenge 1,1-diphenyl-2-picrylhydrazyl (DPPH) radicals under optimal extraction parameters and to compare with traditional extraction solvents. Lastly, Fourier transform infrared (FT-IR) was employed to evaluate the intermolecular interaction of NADES and EA, while scanning electron microscopy (SEM) was applied to characterize the GJ residue before and after extraction with various extraction solvents in order to evaluate the change of surface morphology and the microscopic effect on EA yield.

## 2. Materials and methods

### 2.1. Materials

Whole plants of GJ were purchased from Tongling Hetian Chinese Medicine Pieces Co. Ltd. (Anhui, China) and smashed into powder with a disintegrator (Tianjin Tester Instrument Co, Ltd., Tianjin, China). Analytical grade reagents including choline chloride (≥98% purity), betaine (≥98% purity), oxalic acid (≥98% purity), DL-malic acid (≥98% purity), citric acid (≥98% purity), and malonic acid (≥98% purity) were purchased from Sigma-Aldrich (Shanghai, China). Analytical pure methanol, ethanol, and acetone were purchased from Tianjin Tianli Chemical Reagent Co. Ltd. (Tianjin, China). EA (≥98% purity) was purchased from Sichuan Vicky Biotechnology Co., Ltd. (Sichuan, China). DPPH (≥98% purity) was purchased from Diyibio Biotechnology Co., Ltd. (Shanghai, China). The chromatographic grade methanol was purchased from Honeywell (Arizona, USA). The chromatographic grade phosphoric acid (≥98% purity) was purchased from Tianjin Kemiou Chemical Reagent Co., Ltd (Tianjin, China). Ultra-pure water produced by Milli-Q IQ7000 (Millipore Corp, USA) was used throughout the experiment.

### 2.2. High performance liquid chromatography (HPLC) analysis

**FIGURE 1:**
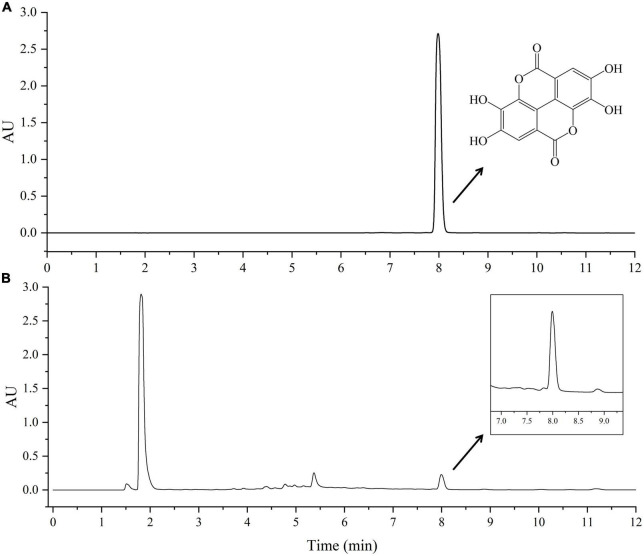
High performance liquid chromatography (HPLC) chromatograms of standard ellagic acid (EA) **(A)** and choline chloride-oxalic acid (ChCl:Oa) extract of *Geum japonicum* (GJ) **(B)**.

The high performance liquid chromatography (HPLC) analysis was performed by Waters Acquity Arc ultra-HPLC system coupled with a photo-diode array detector. The column was Waters SunFire C18 column (4.6 × 150 mm, 5 μm). The mobile phase A was methanol, the mobile phase B was 0.7% phosphoric acid aqueous solution, the flow rate was 1 ml⋅min^–1^, the detection wavelength was 253.4 nm, the column temperature was 30°C, and the injection volume was 10 μL. Gradient elution condition was as follows: 0–5 min, 8∼60%A; 5–10 min, 60∼68%A; 10–11 min, 68∼90%A; 11–12 min, 90∼8%A. The chromatograms of standard EA and choline chloride-oxalic acid (ChCl:Oa) extracted samples were shown in Figure 1. The standard solution of EA was prepared using chromatographic grade methanol and ChCl:Oa. A calibration curve was obtained by injecting standard solutions of EA at different concentrations, i.e., 125, 62.5, 31.25, 15.625, 7.812, and 3.906 μg⋅mL^–1^. The calibration curves were as follows: *Y* = 103883 *X*–38150 (EA in methanol) and *Y* = 103931 *X*–82161 (EA in ChCl:Oa), and the correlation coefficient was 1.000. The relative standard deviation (RSD) of precision, repeatability and stability were 0.40, 2.87, and 0.85%, respectively, and the average recovery of EA was 98.42% and RSD was 2.09%. The matrix effect of EA in the study was lower than 5% in all cases.

### 2.3. Preparation of NADESs

In this work, choline chloride and betaine were selected as HBA, while oxalic acid, DL-malic acid, citric acid, and malonic acid were used as HBD. HBA and HBD were weighted accurately by electronic balance (MS105DU, Mettler Toledo Laboratory, Shanghai, China), mixed in a certain proportion, heated in a 55°C-water bath for 4–6 h (DZKW, Keheng Industrial Development Co., Ltd., Shanghai, China), and stirred until a transparent and uniform liquid was formed. However, betaine-oxalic acid and betaine-citric acid groups could not remain colorless and transparent at room temperature, so they were removed. In total, seven kinds of NADES solvents were synthesized according to the different molar ratios of HBA and HBD in Table 1.

**TABLE 1:** Different types of natural deep eutectic solvents (NADESs).

No.	HBA	HBD	Mole ratio	Abbreviation
A	Choline chloride	DL-malic acid	1:1	ChCl:Ma
B	Choline chloride	Citric acid	1:1	ChCl:Ca
C	Choline chloride	Malonic acid	1:1	ChCl:Mal
D	Choline chloride	Oxalic acid	1:1	ChCl:Oa
E	Betaine	DL-malic acid	1:1	Bet:Ma
F	Betaine	Malonic acid	1:2	Bet:Mal
G	Betaine	Citric acid	1:1	Bet:Ca

### 2.4. NADES-UAE extraction

*Geum japonicum* (∼1 kg) was crushed and sieved through a 50-mesh screen. The fibrous fraction was then crushed and sieved several times. The intact plants of GJ were used as herbs or wild vegetables, so the fine fibers and powder of GJ were mixed well and sealed at room temperature. A certain amount of GJ powder was accurately weighed and put into a 10 ml Eppendorf (EP) tube with 2.5 ml NADES for UAE by an ultrasonic cleaner (KQ-500DE, Kunshan Ultrasonic Instrument Co., Ltd., Kunshan, China). After extraction, the sample was centrifuged (8,000 rpm, 30°C, 20 min), 1 ml of the supernatant was transferred into a 2 ml EP tube, and then 1 ml of ultrapure water was added for dilution. The sample was centrifuged again (10,000 rpm, 20°C, 10 min), and 1 ml of supernatant was taken for HPLC analysis. The standard curve of EA was employed to obtain the EA concentration (mg⋅ml^–1^) which was then converted to mg⋅g^–1^ using Eq. 1:


(1) $$W=\frac{C\times X\times V}{m}$$

C: Sample concentration (mg⋅ml^–1^), X: Dilution ratio, V: NADES volume (ml), m: Dry weight of GJ powder (g), W: EA yield (mg⋅g^–1^).

### 2.5. Single-factor experiment design

The EA yields of different NADESs after extraction were first evaluated under fixed extraction conditions (solid-liquid ratio of 20:1 mg⋅ml^–1^, water content of 30%, extraction temperature of 50°C, extraction power of 300 W, and extraction time of 30 min) to select the best NADES. Subsequently, with the best NADES, the single-factor experiments investigated the effects of water content (30, 40, 50, 60, and 70%), extraction time (10, 20, 30, 40, and 50 min), solid-liquid ratio (5:1, 10:1, 20:1, 30:1, and 40:1 mg⋅ml^–1^), extraction temperature (40, 50, 60, 70, and 80°C), and extraction power (200, 250, 300, 350, and 400 W) on the yield of EA. The extraction conditions for the single-factor experiments were shown in Table 2. Each experiment was repeated in triplicate.

**TABLE 2:** Extraction conditions of single-factor experiments.

Investigate factors	Extracting conditions				
	X_1_ (%)	X_2_ (min)	X_3_ (mg⋅ml^–1^)	X_4_ (°C)	X_5_ (W)
X_1_	–	30	20: 1	50	300
X_2_	50	–	20: 1	50	300
X_3_	50	30	–	50	300
X_4_	50	30	20: 1	–	300
X_5_	50	30	20: 1	50	–

### 2.6. Box-Behnken response surface experimental design

On the basis of BBD principles and single-factor experimental findings, the water content of NADES, extraction time, solid-liquid ratio, extraction temperature, and extraction power were selected as the five BBD factors, and the EA yield was employed as the evaluation index. 46 test sites for response surface testing were designed using a 5-factor and 3-level design method, and the optimal process conditions were determined. Each experiment site was repeated twice, and the average value was calculated and filled in the table. The test factors and level design were shown in Table 3.

**TABLE 3:** Independent variables and levels used for Box-Behnken design (BBD).

Factors	Level		
	−1	0	1
Water content of NADES (X_1_) (%)	40	50	60
Extraction time (X_2_) (min)	20	30	40
Solid-liquid ratio (X_3_) (mg⋅ml^–1^)	10:1	20:1	30:1
Extraction temperature (X_4_) (°C)	50	60	70
Extraction power (X_5_) (W)	250	300	350

### 2.7. Comparison of extraction solvents and extraction methods

The EA yields of NADES-UAE were compared with four conventional extraction solvents-UAE including pure water, methanol, ethanol, and acetone under optimal extraction parameters. In addition, NADES extraction samples under the same conditions were used to compare the EA yields of NADES bath extraction and NADES-UAE. Each experiment was repeated in triplicate.

### 2.8. Determination of EA yield in GJ from different Chinese provinces

Ten batches of GJ from different Chinese provinces (i.e., Henan, Hubei, Zhejiang, Gansu, and Guangdong) were extracted by using the optimized extraction parameters, and the EA yield of each batch of materials was assessed using the previous HPLC method. Each experiment was repeated in triplicate.

### 2.9. Evaluation of DPPH radical scavenging activity

The DPPH radical scavenging assay was performed according to a previously reported method (29). Vitamin C (VC) and EA monomers were dissolved in methanol at concentrations of 10, 20, 40, 60, 80, and 100 μg⋅ml^–1^. Sample concentrations of EA for pure water-UAE, methanol-UAE, ethanol-UAE, acetone-UAE, ChCl:Oa-UAE, and ChCl:Oa extracts of GJ were 39.0625, 78.125, 156.25, 312.5, 625, and 1250 μg⋅ml^–1^. 0.3 mmol⋅L^–1^ DPPH solution was prepared in pure ethanol, and 100 μL of sample solution under optimal extraction parameters was mixed with 100 μL of DPPH solution, allowing to stand in the dark for 30 min. The reaction carrier was a 96-well plate, and the absorbance was measured at 517 nm with an automatic microplate reader (Synergy TM H1, Biotek, USA). The experiments in each group were repeated thrice. VC was used as a positive control. The DPPH radical scavenging rate for each concentration was measured according to the set concentration gradient, and the curves of scavenging rate and sample concentration were plotted, fitted with GraphPad Prism 8 and the half maximal inhibitory concentration (IC_50_) values were calculated. The obtained results were quantified as micrograms of EA equivalents per milliliter of the original GJ sample (μg⋅ml^–1^).

### 2.10. FT-IR of NADES

The Bruker Tensor 27 system was used to record FT-IR spectra (Bruker, Germany). The potassium bromide tablet method was used to prepare the sample. Three samples were prepared: EA monomer, NADES sample, and EA-NADES sample (prepared by dissolving a certain amount of EA in NADES). A quantity of the three samples were taken and mixed with potassium bromide powder, ground and pressed into thin slices for FT-IR measurements. At room temperature, FT-IR measurements were taken in the 400 to 4,000 cm^–1^ scan range.

### 2.11. SEM of GJ residue

Scanning electron microscopy was employed to analyze the morphology of GJ residue. Specifically, the GJ residue was filtered and air-dried naturally after the EA was extracted using the optimized NADES extraction process or other extraction solvents such as methanol, ethanol, and acetone. A few sample particles were placed on a stage, coated with gold palladium, and examined under a SEM (Tescan Vega 3, Tescan Company, China).

### 2.12. Data statistics and analysis

The experimental data was analyzed using SPSS statistical software (Version 23.0). The quantitative data was expressed as mean ± SD and tested using paired *t*-test. Spearman rank correlation analysis was used to investigate the correlation between EA yield and antioxidant activity. *P* < 0.05 indicated statistical significance.

## 3. Results and discussion

### 3.1. Effect of NADES types

The composition of NADESs has a large influence on their physicochemical properties (polarity, viscosity, and solubility), which directly affect the extraction efficiency of the target compounds. According to the previous literature (30, 31), we selected two commonly used HBAs, i.e., choline chloride and betaine, and four HBDs, i.e., DL-malic acid, citric acid, malonic acid, and oxalic acid, which are favorable for phenolic acid extraction. The molar ratios of NADESs were also based on the previous literature (30, 31). NADESs have a large mass transfer resistance, and the excessive viscosity of NADESs at room temperature has a large impact on the EA yield. We devised the following experiment to decrease the viscosity of the solvent system and increase EA yield by adding a specific amount of water.

**FIGURE 2:**
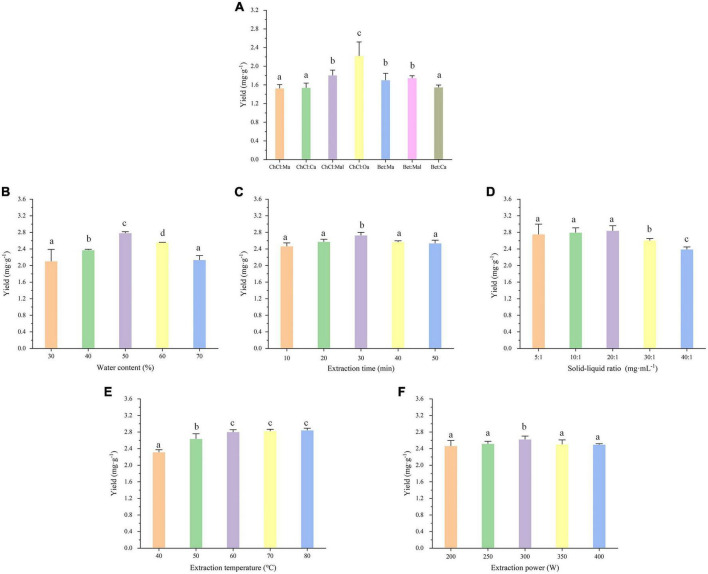
Effects of different natural deep eutectic solvents (NADES) types **(A)**, water content **(B)**, extraction time **(C)**, solid-liquid ratio **(D)**, extraction temperature **(E)**, and extraction power **(F)** on extraction yield of ellagic acid (EA) from *Geum japonicum* (GJ). The extraction solvent choline chloride-oxalic acid (ChCl:Oa) was used to investigate the experimental factors panels **(B–F)**. (Different lowercase letters represent *P* < 0.05, otherwise *P* > 0.05, *n* = 3).

To examine the effects of different NADESs, the initial fixed extraction conditions were as follows: solid-liquid ratio of 20:1 mg⋅ml^–1^, water content of 30%, extraction temperature of 50°C, extraction power of 300 W, and extraction time of 30 min. The results were shown in Figure 2A, indicating that the type of NADES did indeed strongly influence the EA extraction. Among the seven NADESs, ChCl:Oa had the highest yield of EA after extraction. Because oxalic acid had the largest polarity and the strongest acidity among the four HBDs, the formed acidic environment may be beneficial to the extraction and stability of EA. As a result, ChCl:Oa was chosen as the extraction solvent, and we evaluated the effects of numerous parameters on the extraction effect of EA from multiple perspectives.

### 3.2. Effect of NADES water content

The extraction effectiveness of EA was somewhat impacted by the water content of NADES. According to Figure 2B, the EA yield rose across the water content range of 30–50% while declined over the range of 50–70%. The water content of ChCl:Oa was 50%, which resulted in the highest EA production. The presence of a dense hydrogen bond network between the components of ChCl:Oa results in its high viscosity and poor fluidity. Adding the proper amount of water can alter fluidity and mobility to achieve good pore penetration in sample matrices and enhance mass transport from plant matrix to solution (32). Furthermore, the addition of water can increase the polarity of the extraction solvent. When the water content was 50%, the polarity of the extraction solvent may be closer to that of EA molecules, promoting EA dissolution. As a result, proper water content can dramatically improve extraction efficiency. Too much water, on the other hand, may destroy the hydrogen bond of ChCl:Oa and reduce the interaction between ChCl:Oa and EA, lowering extraction efficiency (33).

### 3.3. Effect of extraction time

The extraction time has an effect on the extraction effect as well. It was desirable that obtaining a high EA yield in a short extraction time. As indicated in Figure 2C, increasing the extraction time from 10 to 30 min enhanced the extraction yield. However, the extraction yield decreased gradually as the extraction time increased from 30 to 50 min, which could be attributed to hydrolysis of the target compound under high temperature and long extraction conditions (34). Furthermore, a prolonged extraction time may result in the oxidation of phenolic hydroxyl groups in EA molecules. For this reason, 30 min is the most applicable parameter.

### 3.4. Effect of solid-liquid ratio

The EA yield was impacted by the solid-liquid ratio. Various solid-liquid ratios were investigated, including 5:1, 10:1, 20:1, 30:1, and 40:1 mg⋅ml^–1^, and the results were shown in Figure 2D. The extraction yield increased initially when the solid-liquid ratio was between 5:1 and 20:1 mg⋅ml^–1^ and then declined when it was between 20:1 and 40:1 mg⋅ml^–1^. The optimal solid-liquid ratio was determined to be 20:1 mg⋅ml^–1^, with the maximum extraction yield of EA being 2.8437 ± 0.1085 mg⋅g^–1^. The reason for this may be that the quantitative ChCl:Oa and a small amount of GJ can easily reach the extraction balance. As the solid-liquid ratio increases, the expanded contact area between ChCl:Oa and GJ maybe result in an increase in the solubility of EA. When the solid-liquid ratio was too large, the quantitative ChCl:Oa may not completely extract the EA from GJ, leading to the decrease of EA yield (35).

### 3.5. Effect of extraction temperature

The extraction temperature was the most influential element on extraction efficiency. The effect of extraction temperature on EA extraction yield between 40 and 80°C was investigated, and the results were shown in Figure 2E. The EA yield increased as the temperature rose from 40 to 60°C. Increasing the temperature from 60 to 80°C had a limited impact on the extraction yield. The reason maybe that high temperature tends to reduce these physical adsorption and chemical interactions, which increases the leaching of EA from extraction solutions (36). Moreover, high extraction temperatures can significantly reduce the viscosity of extraction solvents, increase extraction solvent diffusion, and accelerate EA mass transport. When the temperature is too high, EA hydrolysis may reduce its extraction yield, so the EA yield stops growing or starts decreasing (37). Hence, 60°C was believed to be the optimal extraction temperature, as it produced large yields.

### 3.6. Effect of extraction power

The extraction yield was influenced by extraction power. The yield of EA steadily rose as the extraction power increased, as seen in Figure 2F. The yield then peaked at 300 W of extraction power and then progressively started to decline after that. The possible reason was that the increase of extraction power leads to the increase of inter molecular vibration, making EA more easily dissolved into the extraction solvent. In addition, extraction power determines the size of cavitation bubbles generated during ultrasonic extraction. The increased extraction power creates smaller and more cavitation bubbles, which tend to grow and rupture, and generate strong pressure pulses to stir the material and substrate, increasing EA yield. When the extraction power was too high, a great number of bubbles will be generated in the solution. This phenomenon will disperse the energy to the container wall and reduce the energy in the liquid medium and material, with the reduction of EA yield (38).

### 3.7. Statistical analysis and the model fitting

To demonstrate the interactions between five independent variables and determine the ideal extraction conditions, a 46 run BBD with five factors and three levels was carried out. The results were shown in Table 4. Data from Table 4 were subjected to analysis of second-order polynomial regression and analysis of the variance (ANOVA) using Design-Expert 10 software. The linear correlation between the five independent factors and the response variables was shown in Eq. 2. Where Y was the response variable; X_1_, X_2_, X_3_, X_4_, and X_5_ were the water content of NADES, extraction time, solid-liquid ratio, extraction temperature, and extraction power, respectively.


(2) $$Y=2.83-0.0337\,X_{1}+0.0608\,X_{2}-0.0297\,X_{3}+0.2217\,X_{4}$$

$$-0.0173\,X_{5}-0.0059\,X_{1}\,X_{2}-0.0321\,X_{1}\,X_{3}-0.0633\,X_{1}\,X_{4}$$

$$+0.0072\,X_{1}\,X_{5}+0.0831\,X_{2}\,X_{3}+0.0467\,X_{2}\,X_{4}$$

$$+0.0255\,X_{2}\,X_{5}-0.0434\,X_{3}\,X_{4}-0.0110\,X_{3}\,X_{5}-0.0106\,X_{4}\,X_{5}$$

$$-0.1191\,X_{1}^{2}-0.1130\,X_{2}^{2}-0.0204\,X_{3}^{2}+0.0692\,X_{4}^{2}-0.1374\,X_{5}^{2}$$

**TABLE 4:** Box-Behnken design (BBD) matrix and response values for the ellagic acid (EA) yield.

Std.	X_1_	X_2_	X_3_	X_4_	X_5_	Extraction yield (mg⋅g^–1^)
1	−1	−1	0	0	0	2.4762
2	1	−1	0	0	0	2.4573
3	−1	1	0	0	0	2.6776
4	1	1	0	0	0	2.6353
5	0	0	−1	−1	0	2.6712
6	0	0	1	−1	0	2.6389
7	0	0	−1	1	0	3.3142
8	0	0	1	1	0	3.1085
9	0	−1	0	0	−1	2.7175
10	0	1	0	0	−1	2.6553
11	0	−1	0	0	1	2.6018
12	0	1	0	0	1	2.6417
13	−1	0	−1	0	0	2.6629
14	1	0	−1	0	0	2.7352
15	−1	0	1	0	0	2.7963
16	1	0	1	0	0	2.7400
17	0	0	0	−1	−1	2.5117
18	0	0	0	1	−1	2.9933
19	0	0	0	−1	1	2.4236
20	0	0	0	1	1	2.8629
21	0	−1	−1	0	0	2.6781
22	0	1	−1	0	0	2.6574
23	0	−1	1	0	0	2.4426
24	0	1	1	0	0	2.7544
25	−1	0	0	−1	0	2.5986
26	1	0	0	−1	0	2.5608
27	−1	0	0	1	0	3.0945
28	1	0	0	1	0	2.8035
29	0	0	−1	0	−1	2.7173
30	0	0	1	0	−1	2.6204
31	0	0	−1	0	1	2.7068
32	0	0	1	0	1	2.5660
33	−1	0	0	0	−1	2.6115
34	1	0	0	0	−1	2.5147
35	−1	0	0	0	1	2.6650
36	1	0	0	0	1	2.5972
37	0	−1	0	−1	0	2.5980
38	0	1	0	−1	0	2.6670
39	0	−1	0	1	0	2.8918
40	0	1	0	1	0	3.1476
41	0	0	0	0	0	2.9405
42	0	0	0	0	0	2.8435
43	0	0	0	0	0	2.7853
44	0	0	0	0	0	2.8370
45	0	0	0	0	0	2.7965
46	0	0	0	0	0	2.7991

The Table 5 provided a summary of the goodness-of-fit, adequacy, and analysis reports of the models. The reliability of the experimental procedure was demonstrated by the high significance of the regression equation model (*P* < 0.0001). Since the lack of fit values were statistically non-significant (*p* = 0.1175 > 0.05), the regression equation was able to adequately explain the findings and forecast the optimum conditions. The determination coefficient (*R*^2^), adjusted determination coefficient (Adjusted *R*^2^), and coefficient of variance can all attest to the goodness-of-fit (C.V.). The determination coefficient (*R*^2^ = 0.8698) demonstrated that the model could explain 86.98% of the total variation. The adjusted determination coefficient (Adjusted *R*^2^ = 0.7656) illustrated that the model could predict the majority of the variation in extraction yield, and the low coefficient variation value (C.V. = 3.37%) revealed the precision and reliability of the regression model’s experimental values. According to the *p*-value, the linear coefficients X_4_ and quadratic coefficients (X_1_^2^, X_2_^2^, X_4_^2^, X_5_^2^) were especially significant (*P* < 0.01), whereas the linear coefficient X_2_ was significant (*P* < 0.05). The analysis of *F*-value and *P*-value showed that the extraction temperature and extraction duration were the two most influential variables on the extraction yield and the main effect relationships of each factor were: extraction temperature > extraction time > water content > solid-liquid ratio > extraction power.

**TABLE 5:** Analysis of the variance (ANOVA) for the second-order polynomial model.

Source	Sum of squares	df	Mean square	*F*-value	*P*-value
Model	1.4	20	0.0701	8.35	<0.0001
X_1_-water content	0.0181	1	0.0181	2.16	0.1540
X_2_-extraction time	0.0592	1	0.0592	7.05	0.0136
X_3_-solid-liquid ratio	0.0142	1	0.0142	1.69	0.2058
X_4_-extraction temperature	0.7861	1	0.7861	93.69	<0.0001
X_5_-extraction power	0.0048	1	0.0048	0.5703	0.4572
X_1_X_2_	0.0001	1	0.0001	0.0163	0.8994
X_1_X_3_	0.0041	1	0.0041	0.4927	0.4892
X_1_X_4_	0.016	1	0.016	1.91	0.1792
X_1_X_5_	0.0002	1	0.0002	0.0251	0.8755
X_2_X_3_	0.0276	1	0.0276	3.29	0.0816
X_2_X_4_	0.0087	1	0.0087	1.04	0.3177
X_2_X_5_	0.0026	1	0.0026	0.3106	0.5823
X_3_X_4_	0.0075	1	0.0075	0.8958	0.3530
X_3_X_5_	0.0005	1	0.0005	0.0574	0.8126
X_4_X_5_	0.0004	1	0.0004	0.0533	0.8193
X_1_^2^	0.1238	1	0.1238	14.75	0.0007
X_2_^2^	0.1114	1	0.1114	13.28	0.0012
X_3_^2^	0.0036	1	0.0036	0.4348	0.5157
X_4_^2^	0.0418	1	0.0418	4.98	0.0348
X_5_^2^	0.1648	1	0.1648	19.64	0.0002
Residual	0.2098	25	0.0084		
Lack of fit	0.1933	20	0.0097	2.94	0.1175
Pure error	0.0164	5	0.0033		
Cor total	1.61	45			
*R*^2^ = 0.8698	Std. dev. = 0.0916				
Adjusted *R*^2^ = 0.7656	Mean = 2.72				
Predicted *R*^2^ = 0.5054	C.V. % = 3.37				

### 3.8. Optimization of the NADES-UAE procedure

**FIGURE 3:**
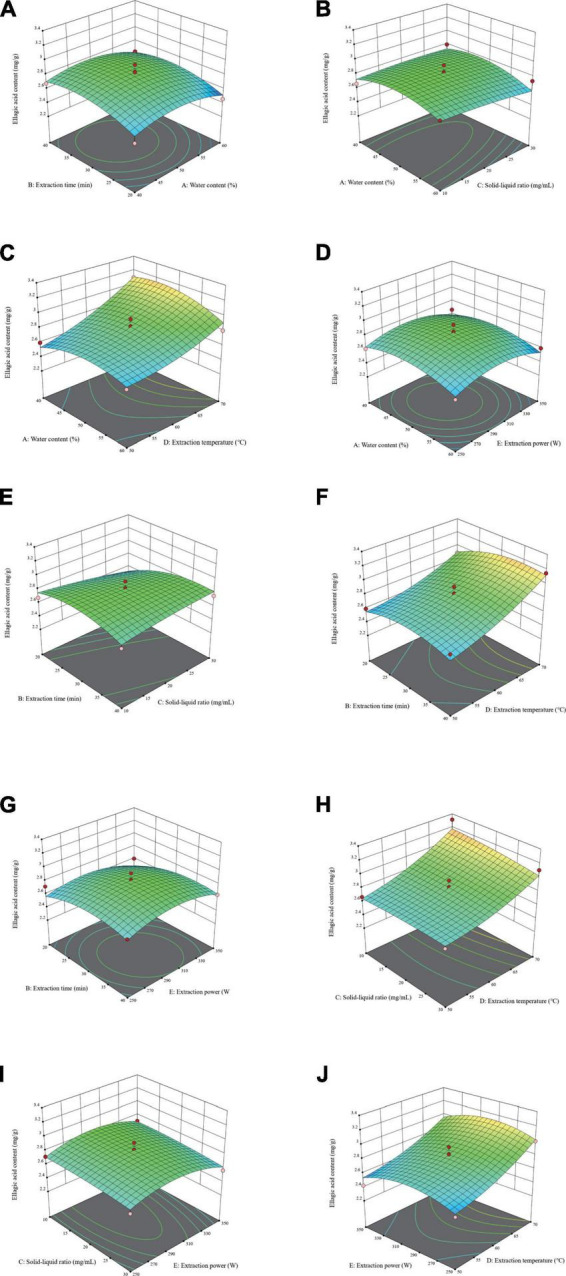
Three-dimensional response surface diagrams of water content of natural deep eutectic solvents (NADES) (X_1_), extraction time (X_2_), solid-liquid ratio (X_3_), extraction temperature (X_4_), and extraction power (X_5_) show the comprehensive effects of experimental factors on extraction yield of ellagic acid (EA). **(A)**: X_1_X_2_, **(B)**: X_1_X_3_, **(C)**: X_1_X_4_, **(D)**: X_1_X_5_, **(E)**: X_2_X_3_, **(F)**: X_2_X_4_, **(G)**: X_2_X_5_, **(H)**: X_3_X_4_, **(I)**: X_3_X_5_, and **(J)**: X_4_X_5_.

As shown in Figures 3A–D, F–J, the three-dimensional response surface plots illustrate the interplay of process factors on response values, and the steepness of the response surface represents the obvious interaction effects. Used in conjunction with the regression equation to explain the response surface graph. The interaction between liquid-solid ratio and extraction duration (X_2_X_3_) had the greatest effect on EA yield, as seen by Table 5 and the degree of curvature in Figure 3E. The yield of EA rose with increasing the solid-liquid ratio in a brief time and decreased with increasing the solid-liquid ratio in a long time, which could be due to the cavitation effect, thermal effect, and mechanical effect of ultrasound. Furthermore, while ultrasonic treatment can encourage EA to penetrate the extraction solution, if the extraction time was too long, high-energy ultrasound waves may destroy EA.

### 3.9. Model validation and method comparison

The software Design-Expert10 was used to determine the optimal values of independent variables and response variables for the proposed extraction: the water content of NADES (X_1_) was 47%, the extraction time (X_2_) was 31 min, the solid-liquid ratio (X_3_) was 10:1 mg⋅ml^–1^, the extraction temperature (X_4_) was 70°C, the extraction power (X_5_) was 300 W, and the predicted highest yield of EA was 3.188 mg⋅g^–1^. Triplicate confirmatory experiments were performed under these optimal extraction parameters, and the EA yield was 3.2142 ± 0.0907 mg⋅g^–1^. The accuracy of the model was calculated and was 99.19% for EA, which indicated the reliability of the models and confirmed that the response model was suitable for optimization.

**FIGURE 4:**
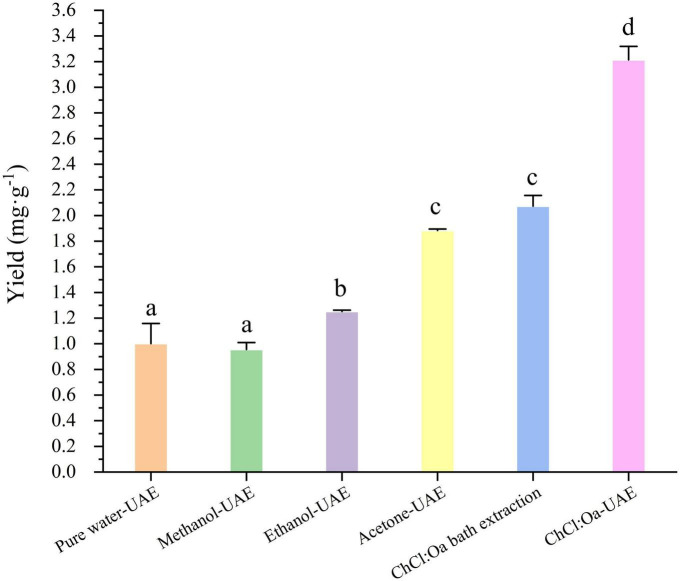
Effects of pure water-ultrasound assisted extraction (UAE), methanol-UAE, ethanol-UAE, acetone-UAE, choline chloride-oxalic acid (ChCl:Oa) bath extraction and choline chloride-oxalic acid-ultrasound assisted extraction (ChCl:Oa-UAE) on ellagic acid (EA) yields. (Different lowercase letters represent *P* < 0.05, otherwise *P* > 0.05, *n* = 3).

The extraction efficiency of different solvents under the same conditions was first compared using the optimized extraction parameters and the results were shown in Figure 4. Due to the low boiling point of acetone solution, the extraction temperature was lowered to 60°C. The EA yield of ChCl:Oa-UAE extraction was significantly higher than that of pure water-UAE, methanol-UAE, ethanol-UAE, and acetone-UAE (*P* < 0.05). The yield of EA extracted with ChCl:Oa-UAE was 3.2 times higher than that of pure water-UAE, 3.3 times higher than that of methanol-UAE and 1.7 times higher than that of ethanol-UAE. The experimental results show that using ChCl:Oa as the extraction solvent can increase the yield of EA. The same NADES was then used to compare the two methods of NADES bath extraction and NADES-UAE. By comparing the EA yield of ChCl:Oa bath extraction with that of ChCl:Oa-UAE, it can be concluded that UAE gave significantly higher EA yields than ChCl:Oa bath extraction (*P* < 0.05), proving that UAE can further improve the extraction efficiency. In conclusion ChCl:Oa has advantages over traditional solvents, and the ChCl:Oa-UAE method is a promising eco-friendly and efficient approach to extract EA from GJ.

### 3.10. EA yield of GJ from different Chinese provinces

Once the appropriate NADES and extraction parameters were determined, they were used to the extraction of GJ samples originating from various geographic regions. According to Table 6, the EA yield varied between 3.0641 ± 0.0767 and 4.0266 ± 0.1292 mg⋅g^–1^. The EA yield of GJ from Henan was significantly higher than the other four provinces (*P* < 0.05). The EA yields of GJ from Hubei, Zhejiang and Guangdong were relatively close, while which of EA in GJ from Gansu was the lowest. The reason may be that the four production areas of Henan, Hubei, Zhejiang, and Guangdong are all located in the eastern part of China and belong to the monsoon region, with abundant rainfall and suitable for plant growth. Gansu is located in the western part of China. It belongs to the non-monsoon region and is a semi-arid region. It has less precipitation and a dry climate, which has a certain impact on plant growth. To sum up, there were certain differences in the EA yield of different batches of GJ in different provinces, which may be related to external growth environmental factors such as humidity, temperature, and soil quality in different production areas. Previous reports have shown that EA exists in a variety of foods such as raspberry and grape, and the EA yield of GJ in this study was slightly higher than that of raspberry (0.2–0.3 mg⋅g^–1^) (39) and closed to grape (1.92–4.31 mg⋅g^–1^) (40). The results shown that GJ was an excellent natural raw material for EA extraction.

**TABLE 6:** Yield of ellagic acid (EA) in *Geum japonicum* (GJ) from different Chinese provinces.

No.	Origin	Batch number	Extraction yield (mg⋅g^–1^)
1	Henan	20190812	3.9553 ± 0.1357^a^
2	Henan	20190316	4.0266 ± 0.1292^a^
3	Hubei	20190601	3.6568 ± 0.0743^b^
4	Hubei	20190812	3.7738 ± 0.1417^b^
5	Zhejiang	20181014	3.6517 ± 0.0631^b^
6	Zhejiang	20190312	3.6774 ± 0.0472^b^
7	Gansu	20190606	3.1997 ± 0.1683^c^
8	Gansu	20190612	3.0641 ± 0.0767^c^
9	Guangdong	20190316	3.5235 ± 0.1104^b^
10	Guangdong	20190812	3.5474 ± 0.0265^b^

Different lowercase letters in the table represent *P* < 0.05, otherwise *P* > 0.05, *n* = 3.

### 3.11. DPPH radical scavenging activity of GJ

**FIGURE 5:**
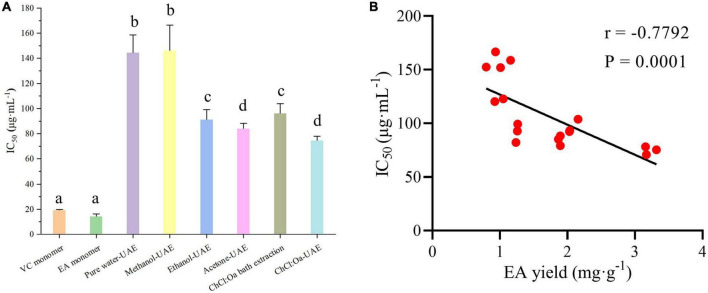
1,1-diphenyl-2-picrylhydrazyl (DPPH) radical scavenging activity of vitamin C (VC) and ellagic acid (EA) monomers and pure water-ultrasound assisted extraction (UAE), methanol-UAE, ethanol-UAE, acetone-UAE, choline chloride-oxalic acid (ChCl:Oa) bath extraction, and choline chloride-oxalic acid-ultrasound assisted extraction (ChCl:Oa-UAE) extracts of *Geum japonicum* (GJ) **(A)** and Spearman rank correlation analysis between EA yield and IC_50_ value **(B)**. (Different lowercase letters represent *P* < 0.05, otherwise *P* > 0.05, *n* = 3, correlation analysis *n* = 18).

1,1-diphenyl-2-picrylhydrazyl is a simple free radical model that can be used to assess the antioxidant activity of samples. As shown in Figure 5A, GJ extract had certain DPPH radical scavenging activity compared with the positive control group. Among all GJ extracts, the ChCl:Oa-UAE had the most pronounced quenching effect in DPPH radical scavenging assay. The DPPH radical scavenging activity of ChCl:Oa bath extract was similar to that of acetone-UAE but significantly higher than that of pure water-UAE and methanol-UAE (*P* < 0.05). Among several conventional extraction solvents, the DPPH radical scavenging activity of acetone-UAE was strongest, followed by ethanol-UAE, and the activity of pure water-UAE was lower than ethanol-UAE and acetone-UAE but slightly stronger than methanol-UAE. The DPPH radical scavenging activity of ChCl:Oa bath extract was comparable to that of ethanol-UAE. Spearman rank correlation analysis was used to explore the relationship between EA yield and IC_50_ value, as shown in Figure 5B. There was a significant correlation between EA yield and IC_50_ value (*P* = 0.0001 < 0.05), and the relationship between EA yield and IC_50_ value was negative (*r* = −0.7792 < 0). So, this above phenomenon can be explained by the different yields of EA in different extracts. Among all the samples, ChCl:Oa-UAE had the highest EA yield, so the DPPH radical scavenging activity was also the strongest.

### 3.12. FT-IR analysis

**FIGURE 6:**
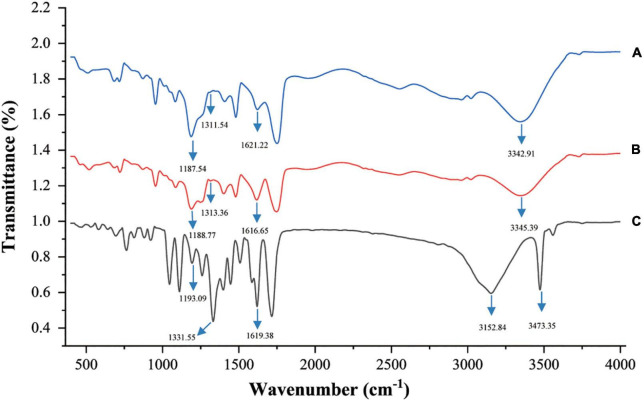
Fourier transform infrared (FT-IR) spectra of choline chloride-oxalic acid (ChCl:Oa) **(A)**, EA-ChCl:Oa **(B)**, and ellagic acid (EA) **(C)**.

To understand the mechanism behind the improved stability of EA caused by ChCl:Oa, intermolecular interactions between EA and ChCl:Oa were investigated using FT-IR (Figure 6). The spectra reveal variation in carbonyl group and C-OH absorption bands from EA in solid and dissolved in ChCl:Oa. The stretching vibration absorption band of EA’s C-OH moved from 1193.09 to 1188.77 cm^–1^ and the deformation vibrational absorption band of EA’s C-OH moved from 1331.55 to 1313.36 cm^–1^, which was coherent with the red-shift in C-OH absorption observed between phenolic compounds and NADES. These results proved the formation of a new hydrogen bond between EA and ChCl:Oa, and also indicated that the conformation of EA in ChCl:Oa was different from that in the solid state (41). Furthermore, the absorption band of C=O in EA decreased from 1619.38 to 1616.65 cm^–1^, indicated that a hydrogen bond exists between the carbonyl group of EA and ChCl:Oa (42). Interestingly, the O-H stretching vibration absorption band of free hydrogen bond in EA was 3473.35 cm^–1^, which became 3345.39 cm^–1^ when EA was dissolved in ChCl:Oa. It is speculated that free OH may form inter molecular hydrogen bond with ChCl:Oa, leading to the change of this absorption band.

### 3.13. SEM analysis

**FIGURE 7:**
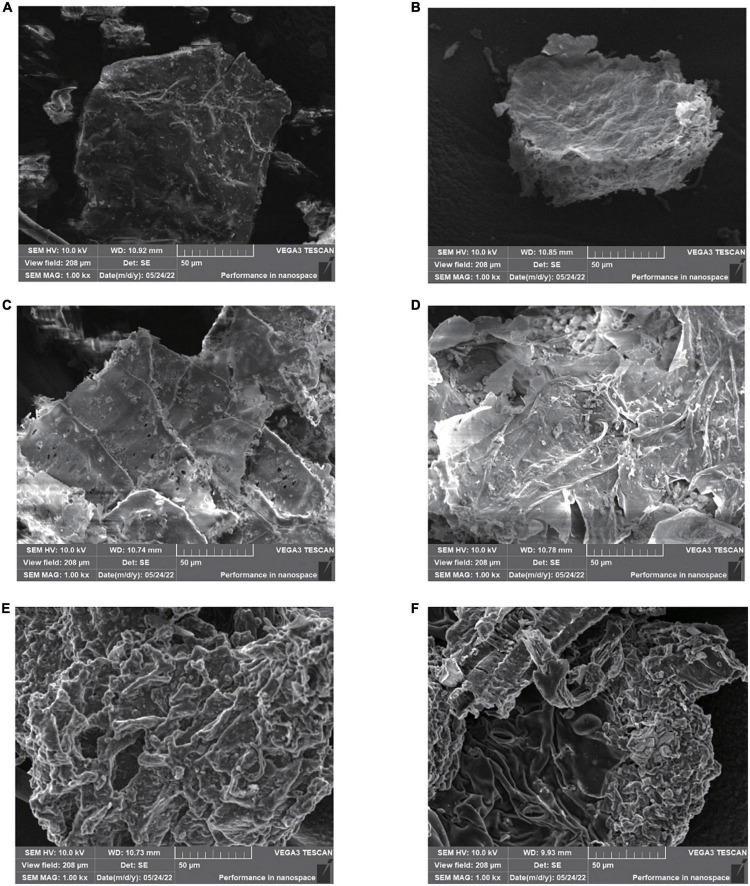
Scanning electron microscopy (SEM) of *Geum japonicum* (GJ) original plant **(A)**, and after extraction with methanol-ultrasound assisted extraction (UAE) **(B)**, ethanol-UAE **(C)**, acetone-UAE **(D)**, choline chloride-oxalic acid (ChCl:Oa) bath extraction **(E)**, and choline chloride-oxalic acid-ultrasound assisted extraction (ChCl:Oa-UAE) **(F)**.

The microstructure of GJ before and after ChCl:Oa-UAE, ChCl:Oa bath extraction and extraction with other solvents was observed by SEM, as shown in Figure 7. It can be seen that the surface structure of the GJ original plant (Figure 7A) was complete and smooth. After organic solvent extraction, the microstructure of GJ changed to some extent. Compared with the original medicinal materials, after extraction with methanol (Figure 7B), GJ was slightly wrinkled but the surface was intact. After extraction with ethanol (Figure 7C), small holes and wrinkles appeared on the surface of GJ. After acetone extraction (Figure 7D), a small part of the surface of GJ was damaged, and the folds were more severe than those of methanol and ethanol. The surface structure and organization of GJ treated with ChCl:Oa bath extraction (Figure 7E) and ChCl:Oa-UAE (Figure 7F) showed obvious shrinkage and became loose and porous. And after ChCl:Oa-UAE extraction, the surface structure and tissue of GJ were destroyed, exposing the internal structure of GJ plant tissue. The comparison results of Figures 7B–E shown that the surface of GJ was severely shrunken after ChCl:Oa bath extraction, indicating the great infiltration effect of ChCl:Oa on the GJ structure. The above phenomenon demonstrates that ChCl:Oa had greater penetration and erosion ability than the other three organic solvents. The significant damage to plant tissue caused by ChCl:Oa-UAE extraction may be a result of the synergistic impact of ultrasonic cavitation and penetration of ChCl:Oa being incorporated into the internal structure of plants (43). Throughout the ChCl:Oa-UAE extraction procedure, the ultrasonic propagation exhibited alternating powerful and weak cycles, leading to the formation of bubbles throughout the disturbance process. As the cavitation effect, the collapse and burst of the bubbles combined by immediate high temperature and pressure accelerate the extraction process (44). In addition, the cavitation action produces a significant shearing force that creates *in-situ* turbulence and destroys the GJ structure. In conclusion, ChCl:Oa-UAE extraction enables ChCl:Oa to be in full contact with surrounding plant tissues, leading to a significant improvement in the extraction efficiency of EA.

## 4. Conclusion

In this study, ChCl:Oa was developed and combined with UAE for the extraction of EA from GJ. The single-factor experiments and RSM were used to optimize the extraction parameters, which were as follows: water content of 47%, extraction time of 31 min, solid-liquid ratio of 10:1 mg⋅ml^–1^, extraction temperature of 70°C and extraction power of 300 W. When comparing the extraction efficiencies of different solvents under the optimized extraction parameters, the EA yield of ChCl:Oa-UAE extract was higher than that of conventional organic solvents. The yield of EA in ten batches of GJ ranged from 3.0641 ± 0.0767 to 4.0266 ± 0.1292 mg⋅g^–1^. According to the DPPH radical scavenging assay, the activity of the ChCl:Oa-UAE extract was greater than that of other traditional solvents such as water and methanol. FT-IR results showed that ChCl:Oa could form a connection with EA through hydrogen bonding, which enhanced the stability of EA. SEM results showed that ChCl:Oa-UAE treatment could destroy the tissue structure of GJ. This study confirmed the synergy between ultrasound as an effective technique and NADES as an alternative solvent for the quick, effective, and environmentally friendly extraction of EA. Hence, these results suggest that the NADES-UAE method can be a green approach for the extraction of EA with high antioxidant activity from GJ.

## Data availability statement

The raw data supporting the conclusions of this article will be made available by the authors, without undue reservation.

## Author contributions

S-JY and Y-PT conceived the experiment and critically reviewed and revised the manuscript. ZY and HG conducted the experiment and drafted the manuscript. QZ and D-QX edited the manuscript. JZ and J-JL revised it. All authors agreed to the version of the manuscript.
